# Supplementary figures and images for: Analysis of differential expression of hair follicle tissue transcriptome in Hetian sheep undergoing different periodic changes
Source: PeerJ. 2024 Nov 25;12:e18542. doi: 10.7717/peerj.18542 (PMC11604043; doi:10.7717/peerj.18542)

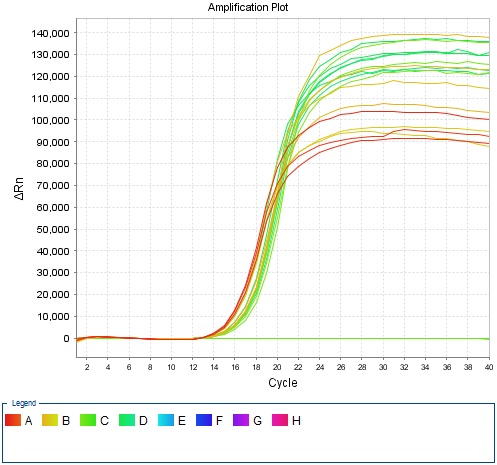

Supplement: Supplemental Information 5 [file peerj-12-18542-s005.zip › Amplification results of qRT-PCR Verification/lncRNA/Amplification Plot-Actin.jpg]

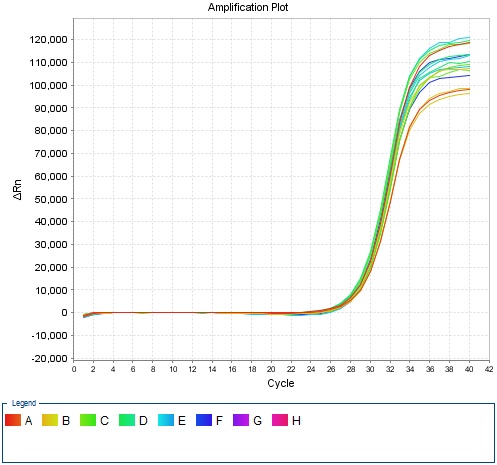

Supplement: Supplemental Information 5 [file peerj-12-18542-s005.zip › Amplification results of qRT-PCR Verification/lncRNA/Amplification Plot-ENSOART00000027222.jpg]

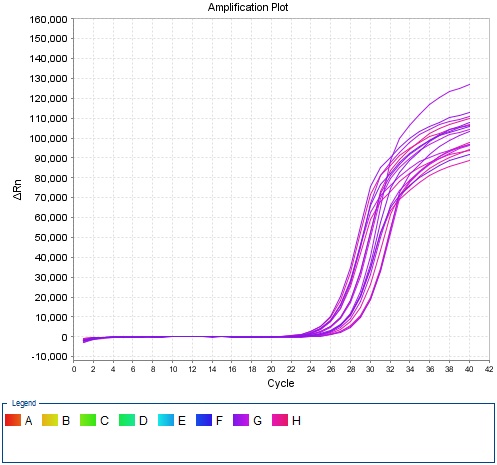

Supplement: Supplemental Information 5 [file peerj-12-18542-s005.zip › Amplification results of qRT-PCR Verification/lncRNA/Amplification Plot-ENSOART00000027625.jpg]

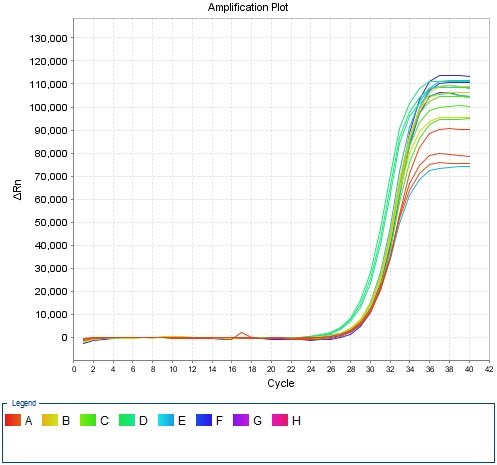

Supplement: Supplemental Information 5 [file peerj-12-18542-s005.zip › Amplification results of qRT-PCR Verification/lncRNA/Amplification Plot-ENSOART00000028006.jpg]

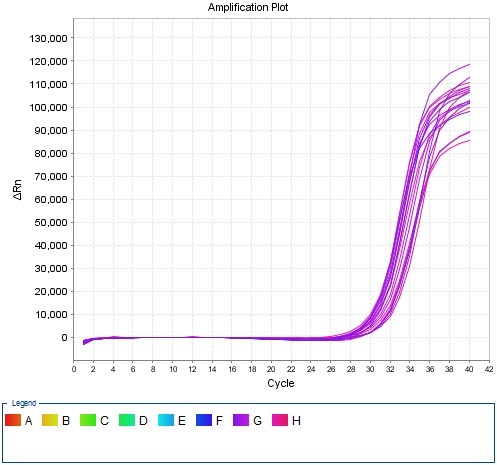

Supplement: Supplemental Information 5 [file peerj-12-18542-s005.zip › Amplification results of qRT-PCR Verification/lncRNA/Amplification Plot-ENSOART00000028189.jpg]

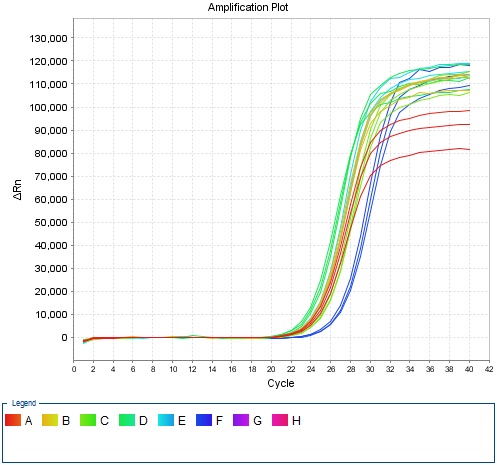

Supplement: Supplemental Information 5 [file peerj-12-18542-s005.zip › Amplification results of qRT-PCR Verification/lncRNA/Amplification Plot-ENSOART00000028369.jpg]

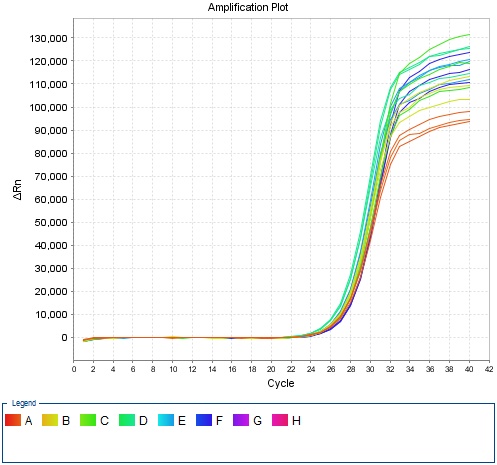

Supplement: Supplemental Information 5 [file peerj-12-18542-s005.zip › Amplification results of qRT-PCR Verification/lncRNA/Amplification Plot-ENSOART00000028374.jpg]

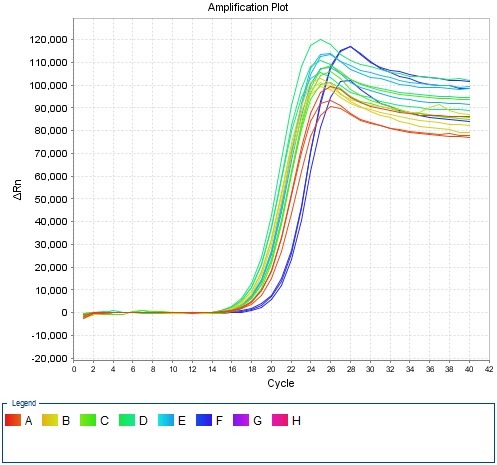

Supplement: Supplemental Information 5 [file peerj-12-18542-s005.zip › Amplification results of qRT-PCR Verification/lncRNA/Amplification Plot-ENSOART00000028675.jpg]

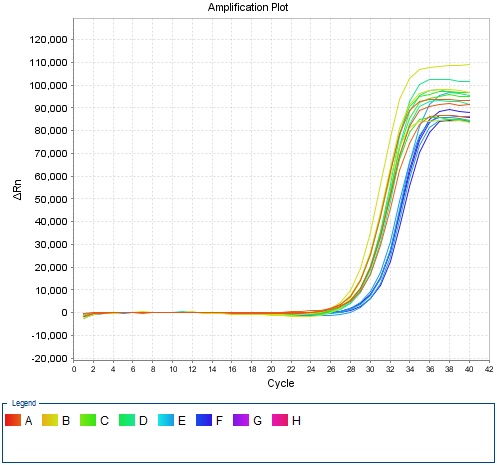

Supplement: Supplemental Information 5 [file peerj-12-18542-s005.zip › Amplification results of qRT-PCR Verification/lncRNA/Amplification Plot-ENSOART00000028779.jpg]

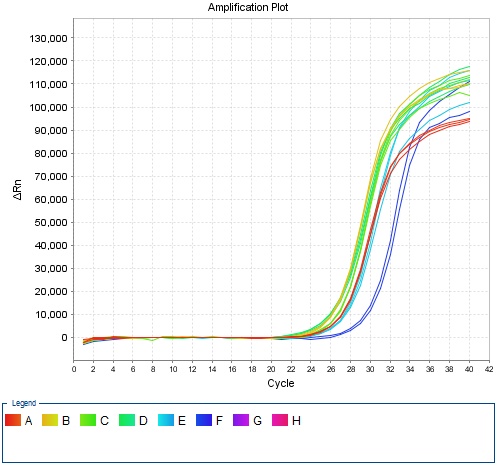

Supplement: Supplemental Information 5 [file peerj-12-18542-s005.zip › Amplification results of qRT-PCR Verification/lncRNA/Amplification Plot-ENSOART00000028803.jpg]

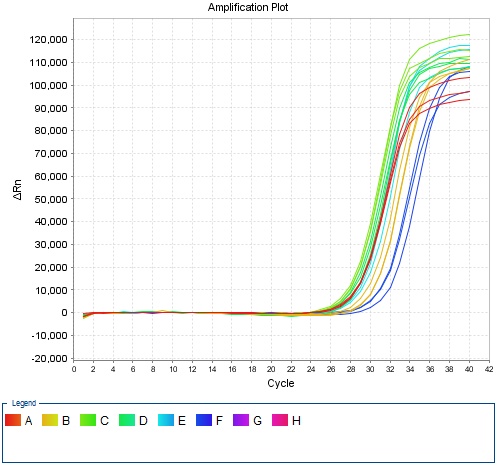

Supplement: Supplemental Information 5 [file peerj-12-18542-s005.zip › Amplification results of qRT-PCR Verification/lncRNA/Amplification Plot-ENSOART00000028961.jpg]

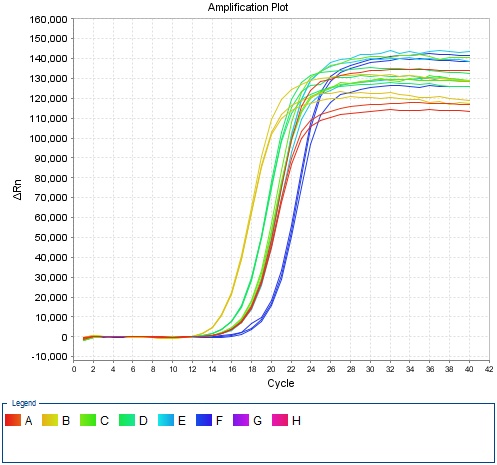

Supplement: Supplemental Information 5 [file peerj-12-18542-s005.zip › Amplification results of qRT-PCR Verification/lncRNA/Amplification Plot-MSTRG.14173.2.jpg]

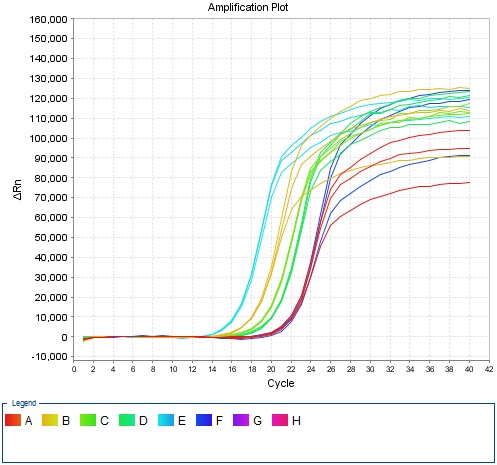

Supplement: Supplemental Information 5 [file peerj-12-18542-s005.zip › Amplification results of qRT-PCR Verification/lncRNA/Amplification Plot-MSTRG.24932.2.jpg]

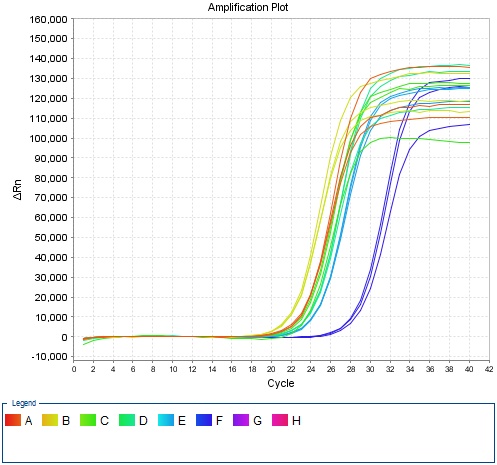

Supplement: Supplemental Information 5 [file peerj-12-18542-s005.zip › Amplification results of qRT-PCR Verification/lncRNA/Amplification Plot-MSTRG.7455.1.jpg]

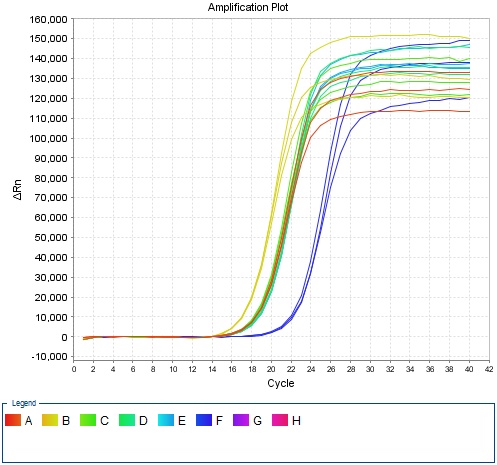

Supplement: Supplemental Information 5 [file peerj-12-18542-s005.zip › Amplification results of qRT-PCR Verification/lncRNA/Amplification Plot3-MSTRG.8053.3.jpg]

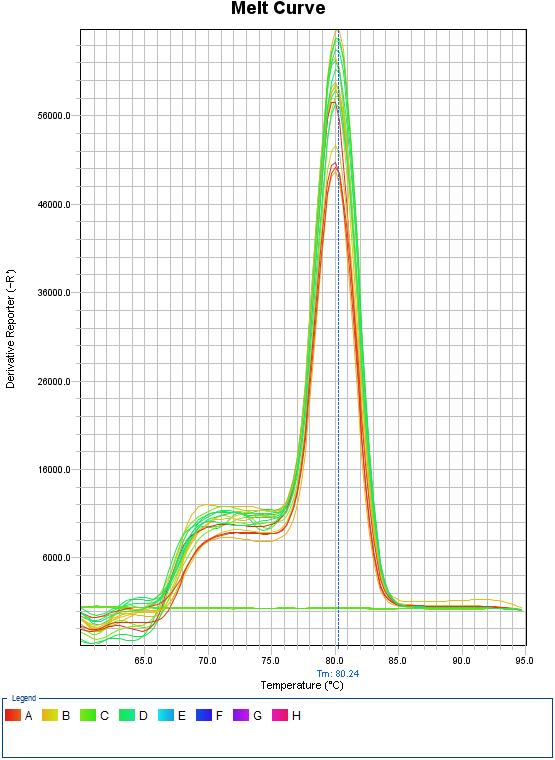

Supplement: Supplemental Information 5 [file peerj-12-18542-s005.zip › Amplification results of qRT-PCR Verification/lncRNA/Melt Curve-Actin.jpg]

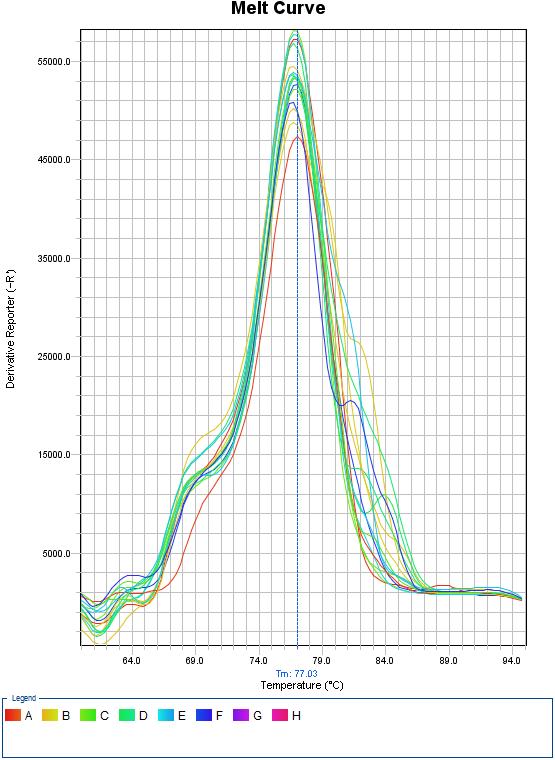

Supplement: Supplemental Information 5 [file peerj-12-18542-s005.zip › Amplification results of qRT-PCR Verification/lncRNA/Melt Curve-ENSOART00000027222.jpg]

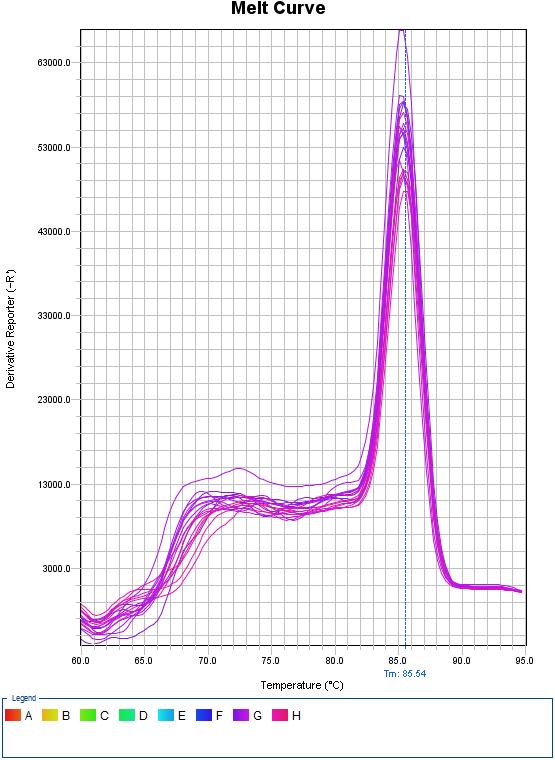

Supplement: Supplemental Information 5 [file peerj-12-18542-s005.zip › Amplification results of qRT-PCR Verification/lncRNA/Melt Curve-ENSOART00000027625.jpg]

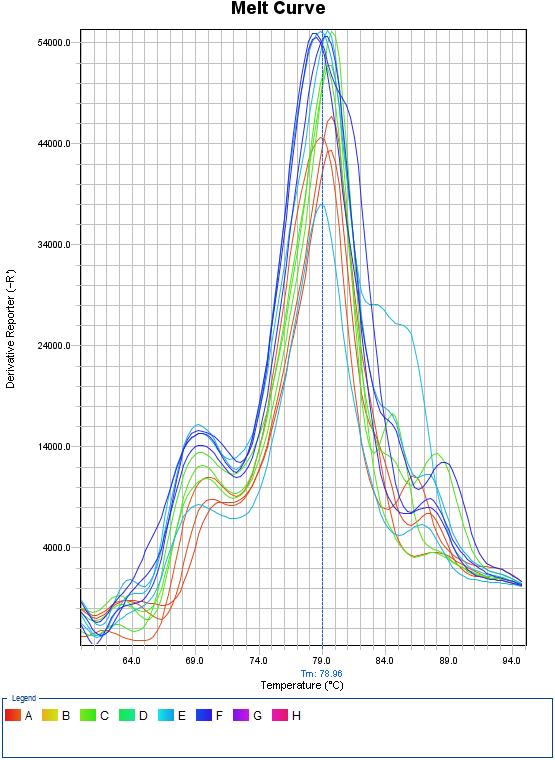

Supplement: Supplemental Information 5 [file peerj-12-18542-s005.zip › Amplification results of qRT-PCR Verification/lncRNA/Melt Curve-ENSOART00000028006.jpg]

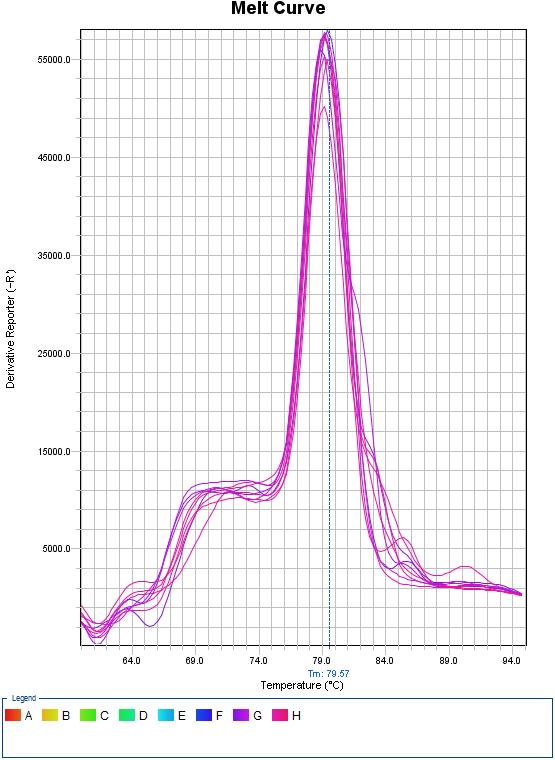

Supplement: Supplemental Information 5 [file peerj-12-18542-s005.zip › Amplification results of qRT-PCR Verification/lncRNA/Melt Curve-ENSOART00000028189.jpg]

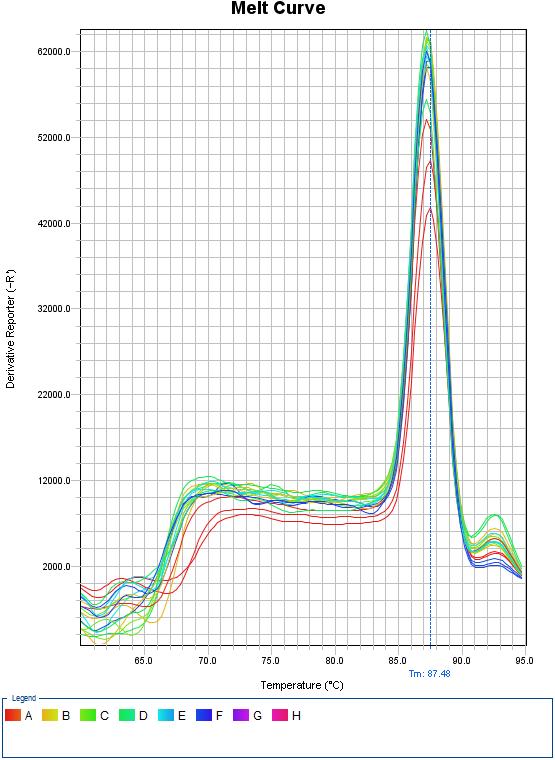

Supplement: Supplemental Information 5 [file peerj-12-18542-s005.zip › Amplification results of qRT-PCR Verification/lncRNA/Melt Curve-ENSOART00000028369.jpg]

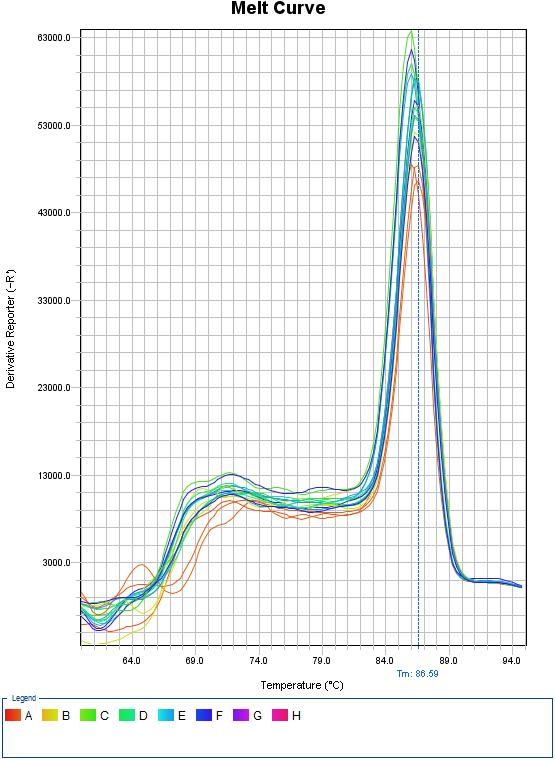

Supplement: Supplemental Information 5 [file peerj-12-18542-s005.zip › Amplification results of qRT-PCR Verification/lncRNA/Melt Curve-ENSOART00000028374.jpg]

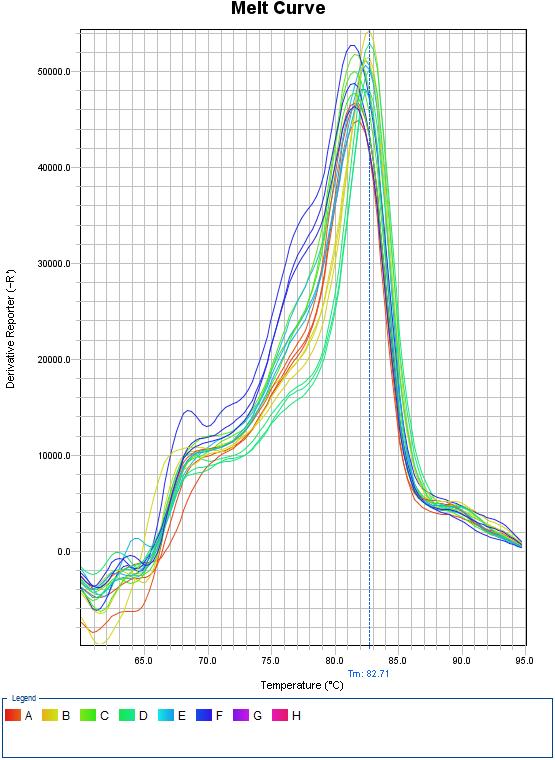

Supplement: Supplemental Information 5 [file peerj-12-18542-s005.zip › Amplification results of qRT-PCR Verification/lncRNA/Melt Curve-ENSOART00000028675.jpg]

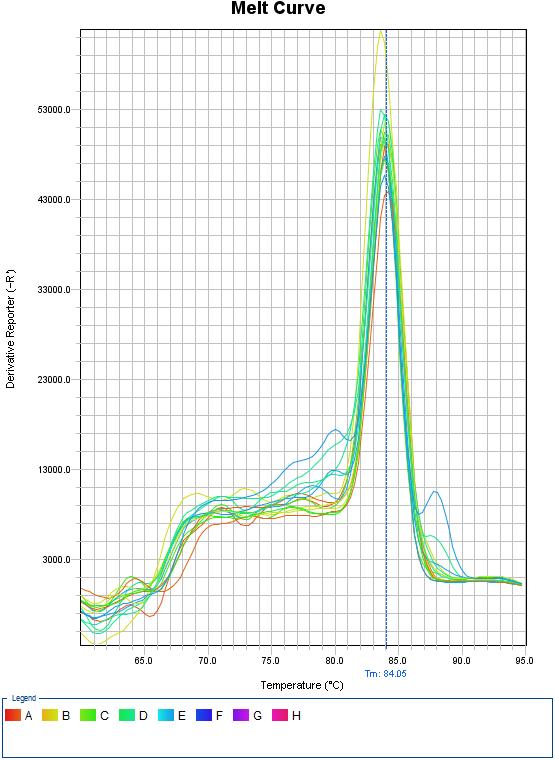

Supplement: Supplemental Information 5 [file peerj-12-18542-s005.zip › Amplification results of qRT-PCR Verification/lncRNA/Melt Curve-ENSOART00000028779.jpg]

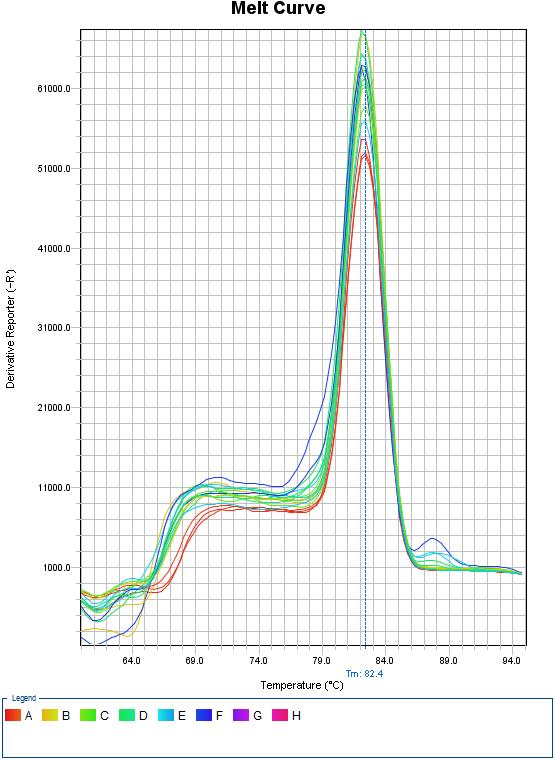

Supplement: Supplemental Information 5 [file peerj-12-18542-s005.zip › Amplification results of qRT-PCR Verification/lncRNA/Melt Curve-ENSOART00000028803.jpg]

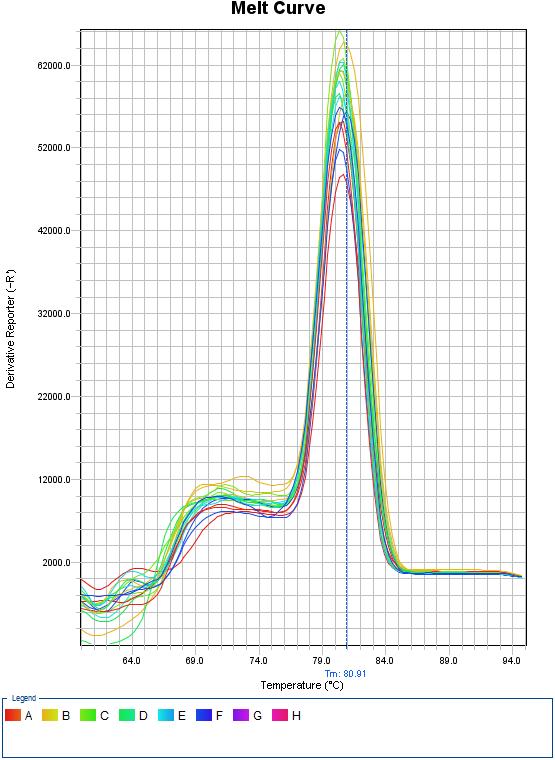

Supplement: Supplemental Information 5 [file peerj-12-18542-s005.zip › Amplification results of qRT-PCR Verification/lncRNA/Melt Curve-ENSOART00000028961.jpg]

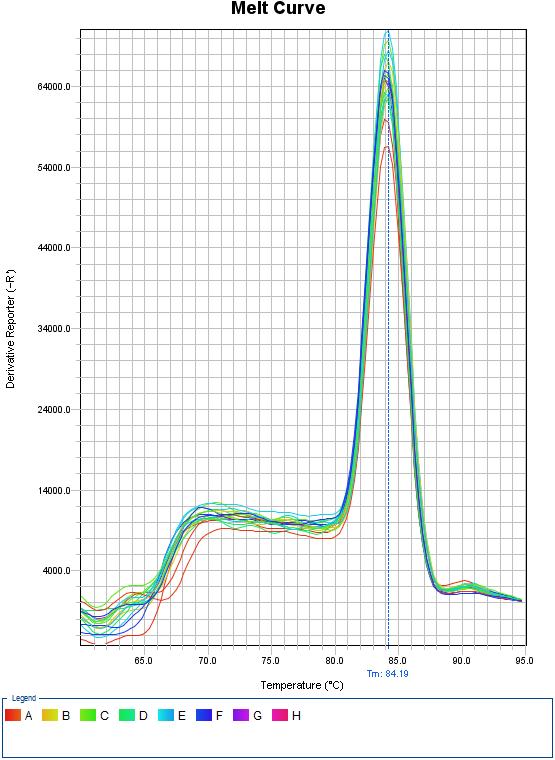

Supplement: Supplemental Information 5 [file peerj-12-18542-s005.zip › Amplification results of qRT-PCR Verification/lncRNA/Melt Curve-MSTRG.14173.2.jpg]

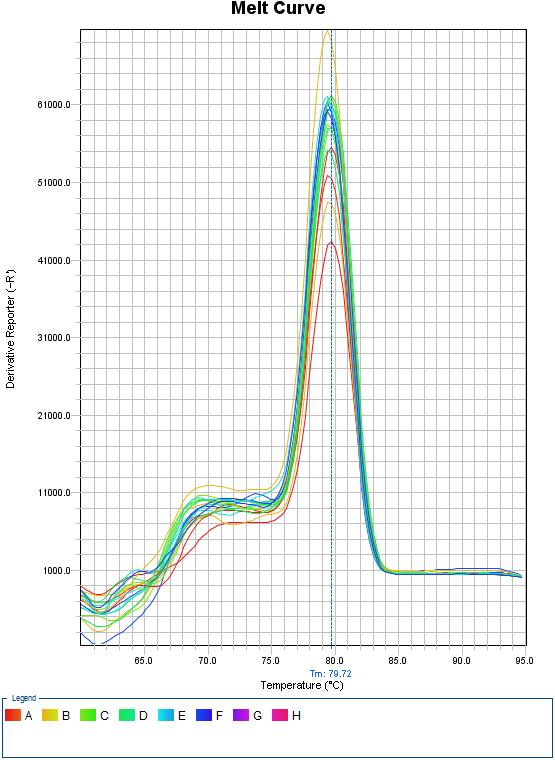

Supplement: Supplemental Information 5 [file peerj-12-18542-s005.zip › Amplification results of qRT-PCR Verification/lncRNA/Melt Curve-MSTRG.24932.2.jpg]

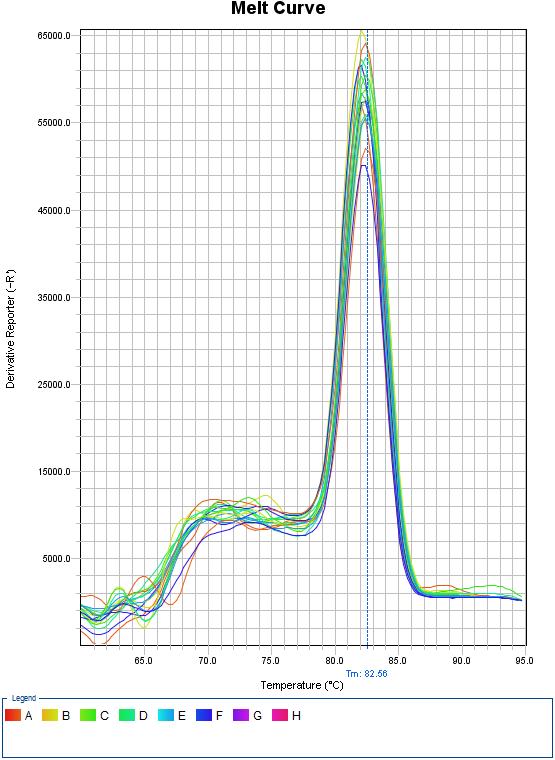

Supplement: Supplemental Information 5 [file peerj-12-18542-s005.zip › Amplification results of qRT-PCR Verification/lncRNA/Melt Curve-MSTRG.7455.1.jpg]

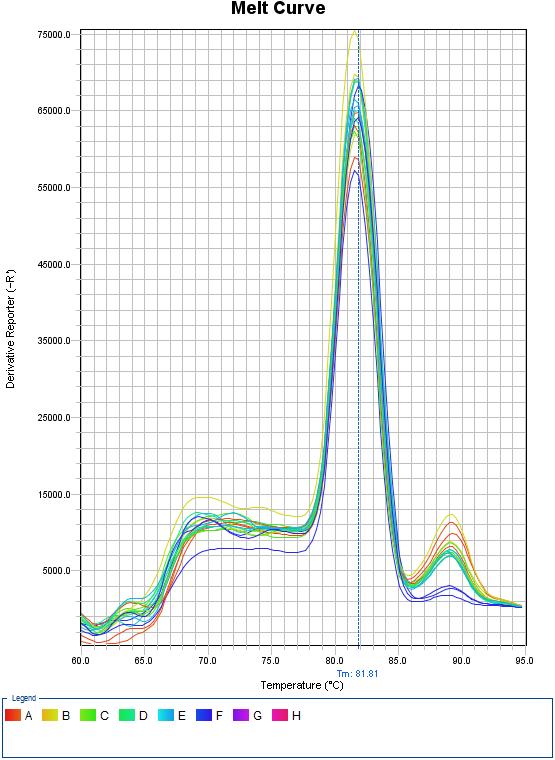

Supplement: Supplemental Information 5 [file peerj-12-18542-s005.zip › Amplification results of qRT-PCR Verification/lncRNA/Melt Curve-MSTRG.8053.3.jpg]

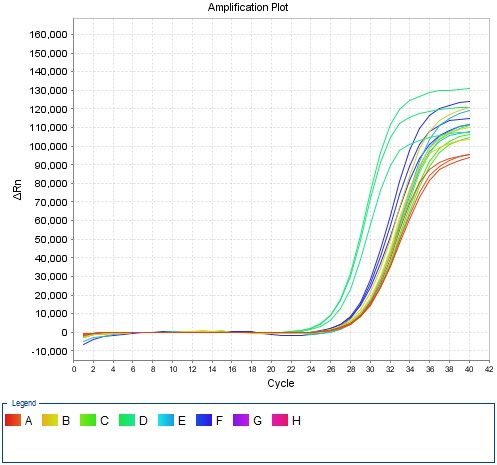

Supplement: Supplemental Information 5 [file peerj-12-18542-s005.zip › Amplification results of qRT-PCR Verification/mRNA/Amplification Plot-ALOXE3.jpg]

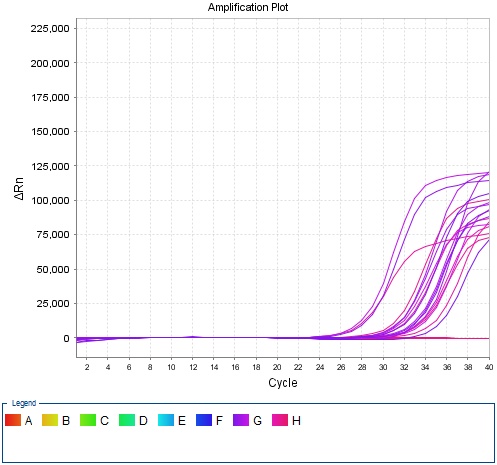

Supplement: Supplemental Information 5 [file peerj-12-18542-s005.zip › Amplification results of qRT-PCR Verification/mRNA/Amplification Plot-AR.jpg]

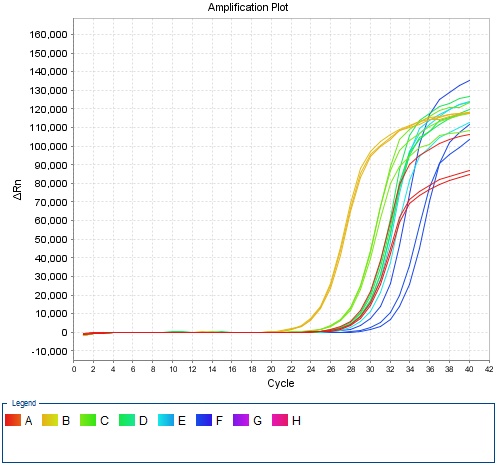

Supplement: Supplemental Information 5 [file peerj-12-18542-s005.zip › Amplification results of qRT-PCR Verification/mRNA/Amplification Plot-CTHRC1.jpg]

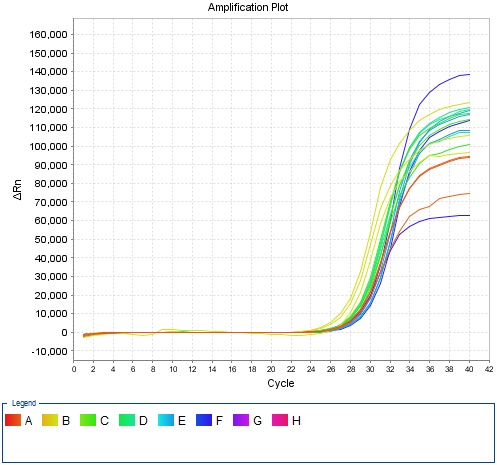

Supplement: Supplemental Information 5 [file peerj-12-18542-s005.zip › Amplification results of qRT-PCR Verification/mRNA/Amplification Plot-CYP24A1.jpg]

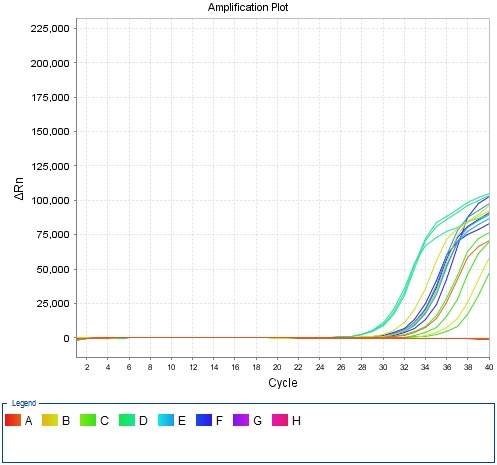

Supplement: Supplemental Information 5 [file peerj-12-18542-s005.zip › Amplification results of qRT-PCR Verification/mRNA/Amplification Plot-HOXA3.jpg]

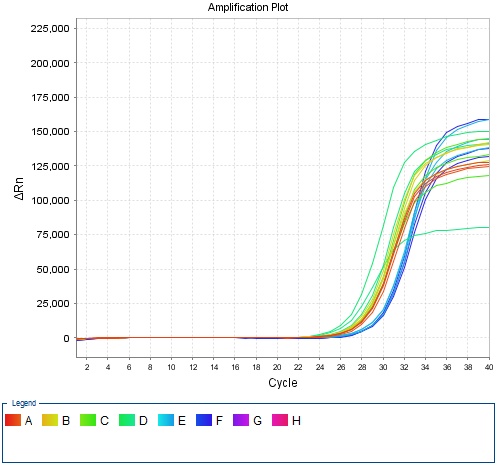

Supplement: Supplemental Information 5 [file peerj-12-18542-s005.zip › Amplification results of qRT-PCR Verification/mRNA/Amplification Plot-KRT84.jpg]

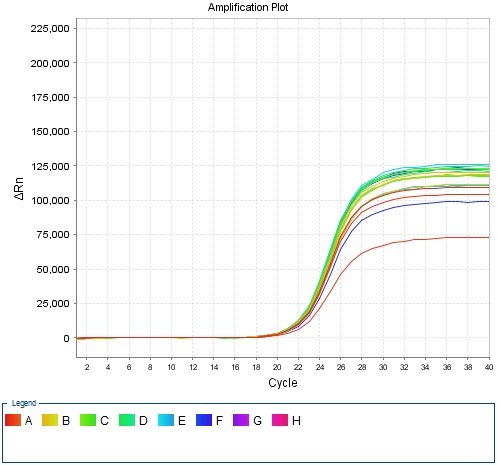

Supplement: Supplemental Information 5 [file peerj-12-18542-s005.zip › Amplification results of qRT-PCR Verification/mRNA/Amplification Plot-LRP2.jpg]

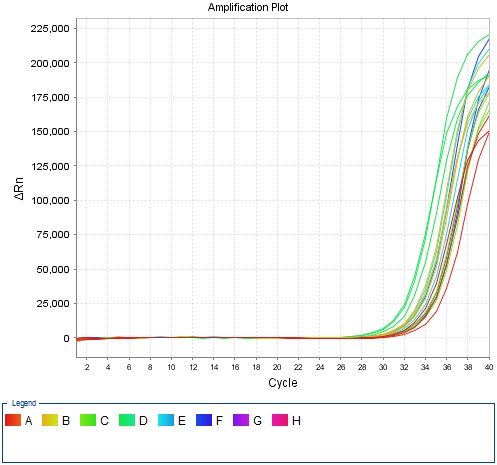

Supplement: Supplemental Information 5 [file peerj-12-18542-s005.zip › Amplification results of qRT-PCR Verification/mRNA/Amplification Plot-PLA2G4D.jpg]

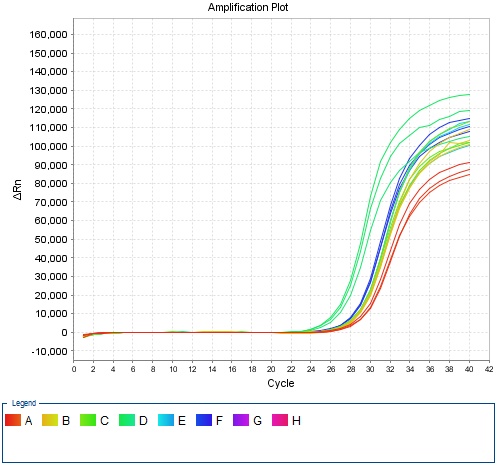

Supplement: Supplemental Information 5 [file peerj-12-18542-s005.zip › Amplification results of qRT-PCR Verification/mRNA/Amplification Plot-PLXNA3.jpg]

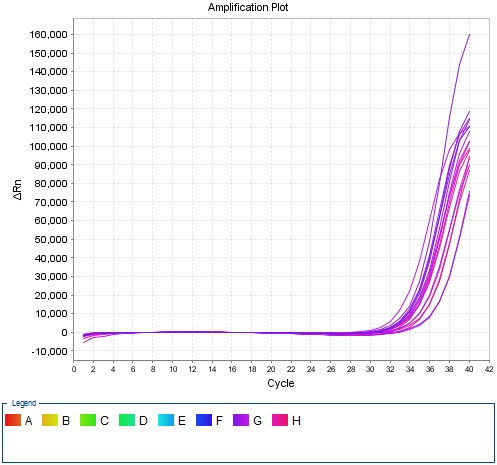

Supplement: Supplemental Information 5 [file peerj-12-18542-s005.zip › Amplification results of qRT-PCR Verification/mRNA/Amplification Plot-RND1.jpg]

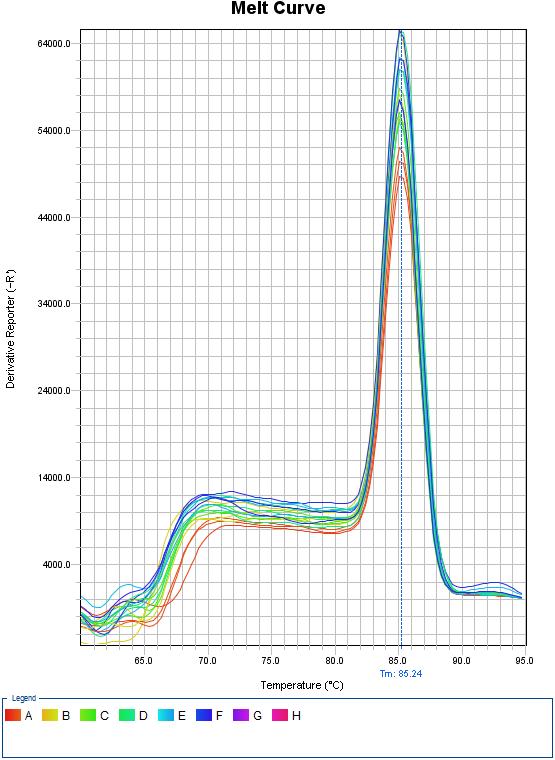

Supplement: Supplemental Information 5 [file peerj-12-18542-s005.zip › Amplification results of qRT-PCR Verification/mRNA/Melt Curve-ALOXE3.jpg]

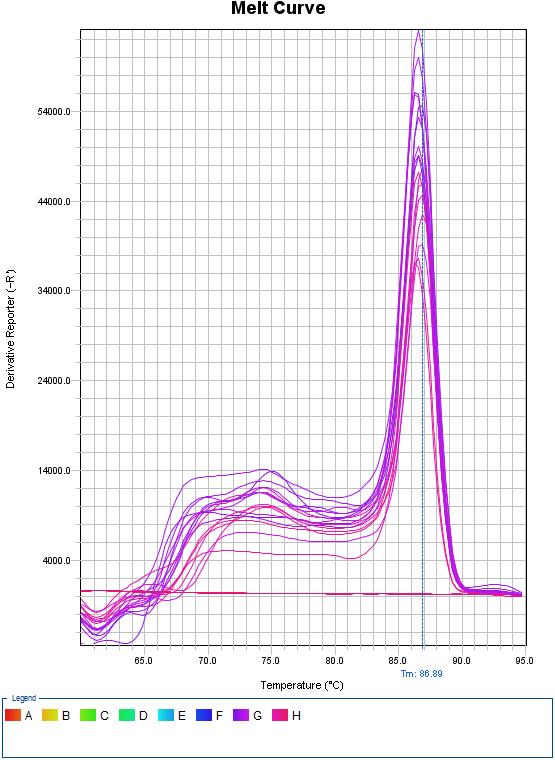

Supplement: Supplemental Information 5 [file peerj-12-18542-s005.zip › Amplification results of qRT-PCR Verification/mRNA/Melt Curve-AR.jpg]

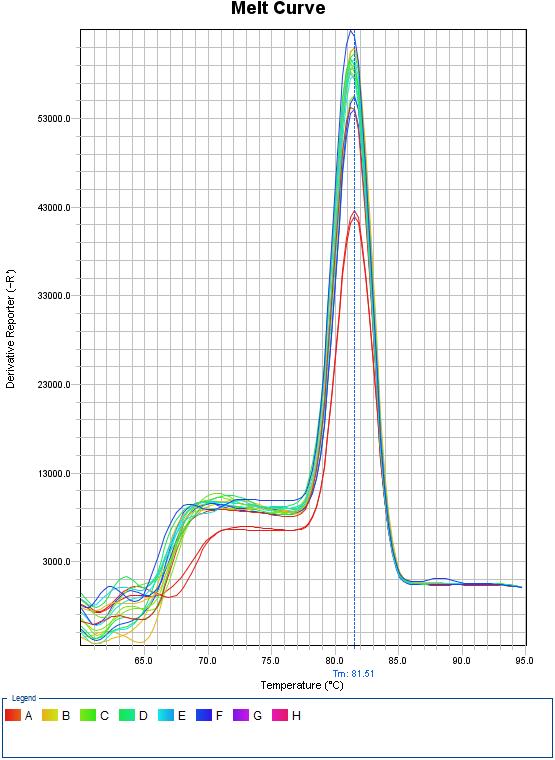

Supplement: Supplemental Information 5 [file peerj-12-18542-s005.zip › Amplification results of qRT-PCR Verification/mRNA/Melt Curve-CTHRC1.jpg]

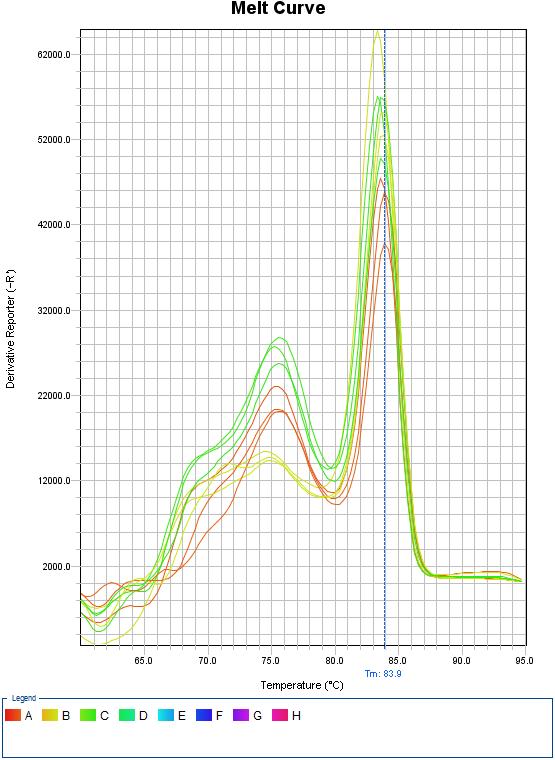

Supplement: Supplemental Information 5 [file peerj-12-18542-s005.zip › Amplification results of qRT-PCR Verification/mRNA/Melt Curve-CYP24A1.jpg]

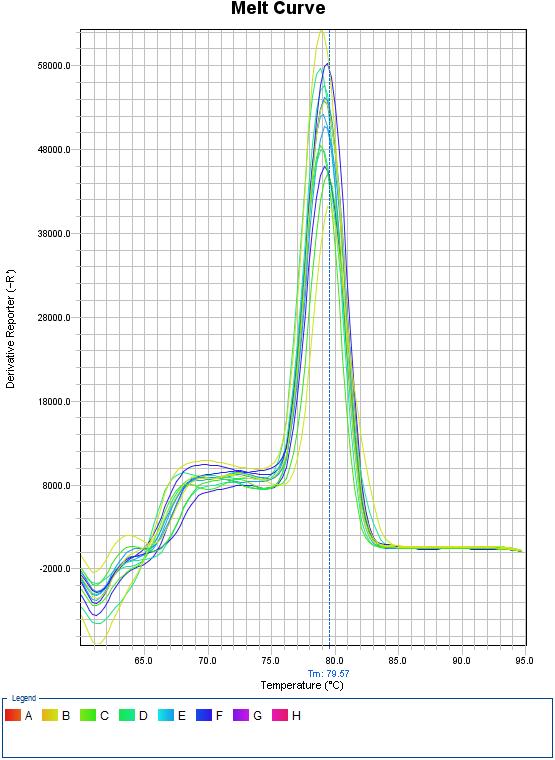

Supplement: Supplemental Information 5 [file peerj-12-18542-s005.zip › Amplification results of qRT-PCR Verification/mRNA/Melt Curve-HOXA3.jpg]

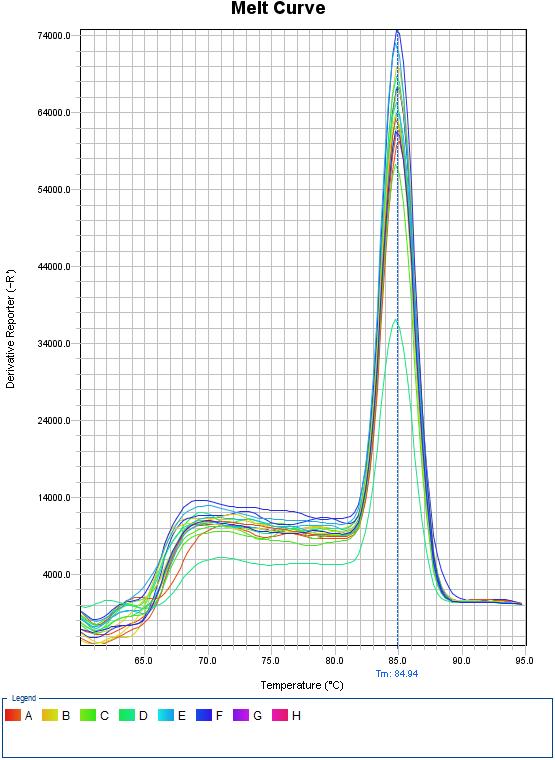

Supplement: Supplemental Information 5 [file peerj-12-18542-s005.zip › Amplification results of qRT-PCR Verification/mRNA/Melt Curve-KRT84.jpg]

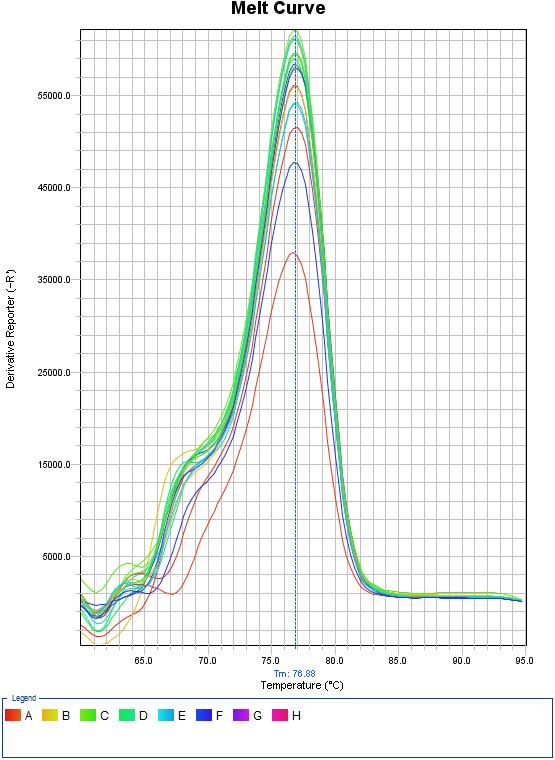

Supplement: Supplemental Information 5 [file peerj-12-18542-s005.zip › Amplification results of qRT-PCR Verification/mRNA/Melt Curve-LRP2.jpg]

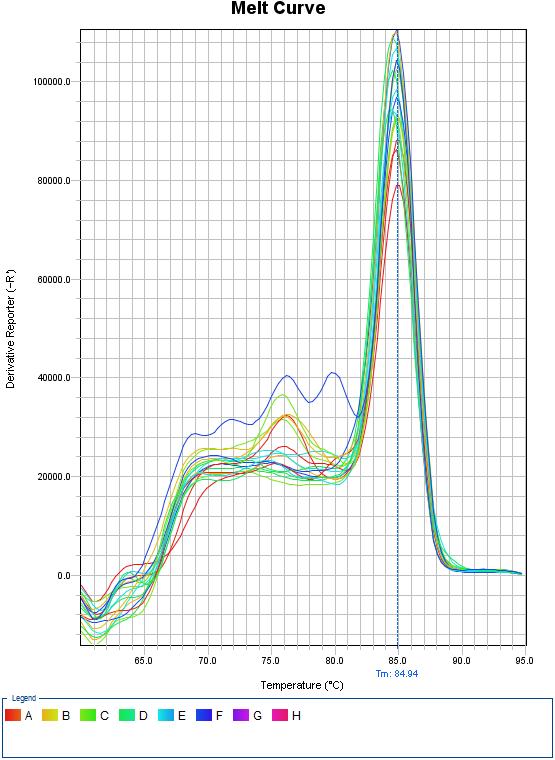

Supplement: Supplemental Information 5 [file peerj-12-18542-s005.zip › Amplification results of qRT-PCR Verification/mRNA/Melt Curve-PLA2G4D.jpg]

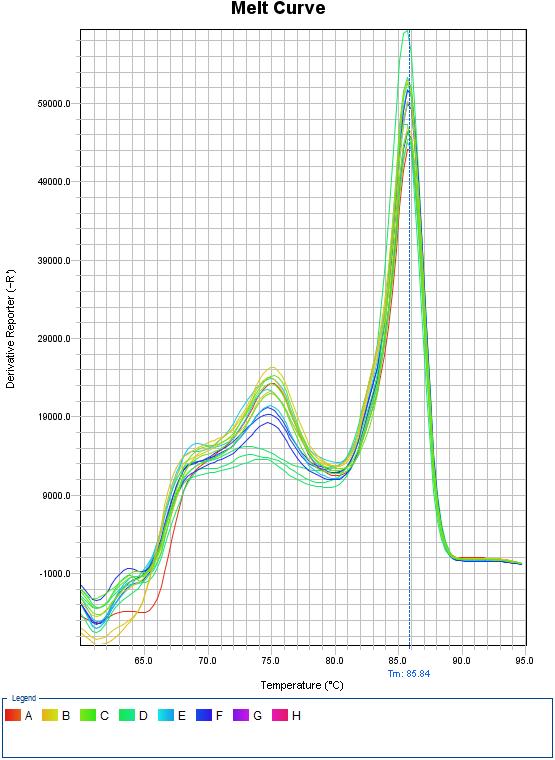

Supplement: Supplemental Information 5 [file peerj-12-18542-s005.zip › Amplification results of qRT-PCR Verification/mRNA/Melt Curve-PLXNA3.jpg]

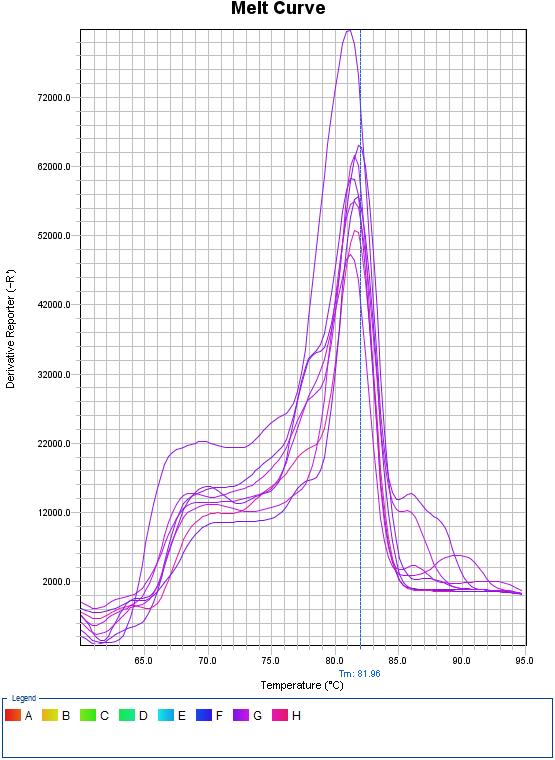

Supplement: Supplemental Information 5 [file peerj-12-18542-s005.zip › Amplification results of qRT-PCR Verification/mRNA/Melt Curve-RND1.jpg]
