# Supplementary material for: Analysis of differential expression of hair follicle tissue transcriptome in Hetian sheep undergoing different periodic changes
Source: PeerJ. 2024 Nov 25;12:e18542. doi: 10.7717/peerj.18542 (PMC11604043; doi:10.7717/peerj.18542)
Supplement: Table S1 [file peerj-12-18542-s006.docx]

Supp. Table 1. The quality control results for the sequencing data

| **Sample** | **index** | **The quantity of data（Gb）** | **Reads（M）** | **Q30（%）** |
| --- | --- | --- | --- | --- |
| **01180635I** | TCCTCATG | 12.326 | 82.18 | 94.2 |
| **01180635II** | GTCCTAAG | 12.746 | 84.98 | 92.96 |
| **01180635III** | CTTAGGAC | 12.993 | 86.63 | 93.08 |
| **00617149I** | TCCACGTT | 13.647 | 90.98 | 92.88 |
| **00617149II** | GTCAGTCA | 14.47 | 96.47 | 94.76 |
| **00617149III** | CGAATACG | 12.955 | 86.37 | 94.37 |
| **01180656I** | TCTAGGAG | 13.759 | 91.73 | 94.41 |
| **01180656II** | CGCAACTA | 12.923 | 86.16 | 94.17 |
| **01180656III** | CTTCACTG | 9.521 | 63.48 | 92.89 |
| **01180656III（Supp）** | CTTCACTG | 2.894 | 19.3 | 94.39 |

Note：Q20=bases of Q>=20 / all bases of sequencing，Q30=bases of Q>=30 / all bases of sequencing.
