# Supplementary material for: Analysis of differential expression of hair follicle tissue transcriptome in Hetian sheep undergoing different periodic changes
Source: PeerJ. 2024 Nov 25;12:e18542. doi: 10.7717/peerj.18542 (PMC11604043; doi:10.7717/peerj.18542)
Supplement: Table S2 [file peerj-12-18542-s007.docx]

Supp. Table 2. mapping_statistic

| **sample** | **total reads** | **mapped reads** | **pair mapped reads** | **single mapped reads** | **mapped ratio** |
| --- | --- | --- | --- | --- | --- |
| **S00617149I** | 89759340 | 89563577 | 89467396 | 96181 | 99.78% |
| **S00617149II** | 90462322 | 90201596 | 90077628 | 123968 | 99.71% |
| **S00617149III** | 84823360 | 84681761 | 84607162 | 74599 | 99.83% |
| **S01180635I** | 79434522 | 79321392 | 79255206 | 66186 | 99.86% |
| **S01180635II** | 83198370 | 83085828 | 83027818 | 58010 | 99.86% |
| **S01180635III** | 85629238 | 85437102 | 85347122 | 89980 | 99.78% |
| **S01180656I** | 87969762 | 86940432 | 86475170 | 465262 | 98.83% |
| **S01180656II** | 84280136 | 84171871 | 84107788 | 64083 | 99.87% |
| **S01180656III** | 62064902 | 61931959 | 61874880 | 57079 | 99.79% |
