# Supplementary material for: Analysis of differential expression of hair follicle tissue transcriptome in Hetian sheep undergoing different periodic changes
Source: PeerJ. 2024 Nov 25;12:e18542. doi: 10.7717/peerj.18542 (PMC11604043; doi:10.7717/peerj.18542)
Supplement: Table S3 [file peerj-12-18542-s008.docx]

Supp. Table 3 The parameters of the primer sequences for qRT-PCR Verification

| Gene Name | Primer Sequences (5′–3′) |
| --- | --- |
| PLA2G4D-F | GATGACGCCTGGTTCAAGGT |
| PLA2G4D-R | AGGAACTCCACGTCCAGCTC |
| LRPⅡ-F | GCGGAGCCTGCTATAACACC |
| LRPⅡ-R | TCAGTGCGCGAGCATAGGT |
| KRT84-F | AAGTGGTCTCGGCTATGGCT |
| KRT84-R | ATTGGGGTCAATCTCCAGGT |
| HOXA3-F | TTTGATCGTGGGATCTGTAGC |
| HOXA3-R | TCTGACCACCATCTGGAAAAC |
| AR-F | CAGCTGCTCCACCGATCTTA |
| AR-R | GGTCGAACTGCCTCCTAGGT |
| CTHRC1-F | TCAGCGGCTCACTTCGGT |
| CTHRC1-R | GGGCTTCCTTGGTCCAAATA |
| PLXNA3-F | TGTGGACGGCAAGTCTGAGT |
| PLXNA3-R | TGATCTGCGAGGAGACGAACT |
| ALOXE3-F | CTACTCCGCCACCAAGACG |
| ALOXE3-R | CCGGATGTCATCCAGCTTCT |
| CYPⅡ4A1-F | GAAGGCCTACCGCGACTATC |
| CYPⅡ4A1-R | TCAGCCAAGACCTCGTTGAT |
| RND1-F | GGTACGTCCAAGGTGTGAACAG |
| RND1-R | GTCCCAGAGACTGAGCTCCAC |
| MSTRG.24932.2-F | TTTACCCCATGGCTTAGGTTC |
| MSTRG.24932.2-R | ATCAAGGGGCATTCTGGCT |
| MSTRG.14173.2-F | AGGCAGCGGAGGCAGACT |
| MSTRG.14173.2-R | ATGGGACAGCTCCACCTTCA |
| MSTRG.8053.3-F | GGAGGATTGGCCAGATCACT |
| MSTRG.8053.3-R | AACGCACTAACGGAAAAGACA |
| MSTRG.7455.1-F | GGAACTCAGGAGCCGATTCT |
| MSTRG.7455.1-R | ACAAGAGATGGGGGCAGCTA |
| *ENSOART00000027625-F* | CTGAGGAGCTGGAGGCCTTA |
| *ENSOART00000027625-R* | TTTCCTCCAGGTGTGGTCAAT |
| *ENSOART00000028803-F* | AGCCTCGACTAGACGGACCT |
| *ENSOART00000028803-R* | TTGCCACGAAGTGATGGAAC |
| *ENSOART00000028006-F* | ACTGCAGAAGCCGCTGGAC |
| *ENSOART00000028006-R* | GGGTTTCAGCACAGCCTCAG |
| *ENSOART00000028189-F* | ACACGTTAGCCGGCCATC |
| *ENSOART00000028189-R* | TGAGGCTTGTCAAATGCTTCA |
| *ENSOART00000028961-F* | CAGCTGGAACCTCCTTGAGTC |
| *ENSOART00000028961-R* | CACGCACGAAGCTAAAGCTC |
| *ENSOART00000027222-F* | TGGTTCCTGGTGCAGTGTCT |
| *ENSOART00000027222-R* | GCCCACAGCACCTCAATTCA |
| *Actin-F* | AAGGCCAACCGTGAGAAGAT |
| *Actin-R* | CGTCACCGGAGTCCATCAC |
